# Sycosis

**Published:** 1887-03

**Authors:** Julius Schmitt

**Affiliations:** Rochester, N. Y.


### "SYCOSIS."

JULIUS SCHMITT, M. D., ROCHESTER, N. Y.

Sycosis is one of the heads of Hahnemann's hydra of chronic diseases. The name is derived from δυκοτ, a "fig," and has reference to fig-warts. The old school understands under Sycosis a folliculitis affecting the beard and the scalp. According to Hahnemann, this disease is caused by a miasma, which shows itself at first as a gonorrhœa, but attacks the entre organism like syphilis. He further says that this gonorrhœa is quite different from the local irritation that causes a simple urethritis; and he makes, therefore, a distinction between a mild urethritis and sycotic gonorrhœa, analogous to that between a chancroid and a chancre.

The master, who gave us the symptoms of the psora-disease, had to leave to his true followers the work of enlarging our knowledge on the two other miasmata, viz.: syphilis and sycosis. The former has been partially described by old-school authorities, yet the symptoms and pathological conditions caused by syphilis are mixed with those due to mercurial poisoning to such a degree, that the differentiation between hydragyrosis and syphilis is almost impossible. The latter, sycosis, found its best investigator in Von Bœnninghausen. He gave a full statement of its symptoms in his "Anamnesis of Sycosis," a translation of which you will find on page 321 of the third volume of THE HOMŒOPATHIC PHYSICIAN, and he also claims that sycosis is propagated, not only by the specific gonorrhœa of Hahnemann, but also by small-pox and vaccine virus, that is, that these three poisons are identical.

A good many observations made by pure homœopaths point to the correctness of Bœnninghausen's views.

Is it not peculiar that Thuja cures gonorrhœal discharges and at the same time small-pox? I remind you here of the variola epidemic that raged in 1871 in Philadelphia, where Thuja, corresponding to the Spiritus epidemicus, cured very many cases, and that without pitting. Have you not seen Antimonium tartaricum cure sycotic-chancre and small-pox?

Is not Silicea indicated in a purulent, badly smelling gonorrhœa, and does it not also cure bad effects from vaccination?

Many more examples might be given, but these may suffice.

Now, gentlemen, if Bœnninghausen's propositions are true, sycosis becomes a very important object for our study, and with your permission I shall try to give you a short description of acute and chronic sycosis and its treatment.

Sycosis, like syphilis, is the consequence of an impure coitus. After the exposure, a time of incubation, varying from seven to fourteen days, follows. Yet cases have been reported where symptoms of general malaise were noticed the very next day, although it took several days for the critical discharge to make its appearance. In the beginning, the discharge will not differ much from that we meet in a simple urethritis; perhaps the local symptoms may be very slight indeed, while in urethritis we generally find a highly inflammatory condition. The general feeling of the patient, however, will point to a disturbance of the whole system which is out of proportion to the local trouble. There may be mental depression, sleeplessness, general debility, inappetency, or canine hunger, bad taste in the mouth, constipation or diarrhœa, and so on. The discharge, at first mucous, gradually grows thick, purulent, green or brown, and offensive, and fig-warts may develop on the genitals.

If the patient is not cured in this condition (which in the majority of cases does not happen, as the pure Hahnemannian is the only one that is able to do so) and the symptoms are suppressed by local means or other unscientific procedures, the disease will become chronic. There may follow an apparent interval of health, but, in closely examining such a patient, you will find some symptoms, being perhaps too trifling to be complained of, which, however, show the existence of a condition not corresponding with the perfect health he may have enjoyed before the gonorrhœal attack.

These slight symptoms may remain stationary or increase very gradually, according to the stronger or weaker life-force which holds them in abeyance, but, nevertheless, a time will come when this life-force loses its strength or becomes undermined, either by an acute attack of sickness or great mental disturbance, and then the sycotic miasma will no longer remain latent, but break out in full force and kill its victim, unless the science of Homœopathy comes to the rescue.

The prognosis in this disease is favorable if the proper homœopathic remedy is selected. Hahnemann's treatment of the acute attack was very simple, and he generally found Thuja and Nitric acid, given according to their indications, sufficient for a cure *if the patient came under treatment before a latent miasma like psora or syphilis had been aroused.*

But, unfortunately, these simple cases are rather the exception than the rule, and Thuja or Nitric acid, to which we will add, right here, Phosphoric acid and Sabina, will have to be complimented by some anti-sporic or anti-syphilitic remedy.

In chronic sycosis the treatment grows still more difficult, since then the patient will almost always present sycotic and psoric symptoms, and the worst cases will be those where syphilis is combined with the other two.

We shall have to find, by the most careful examination of the patient, which of the two or three miasmata has to be attacked first, and which next, and a correct alternation (always in the sense of Hahnemann) of anti-sycotics, anti-sporics, and anti-syphilitics may lead us finally to a cure if nature has preserved enough reactive power and the patient has not been abused by unscientific drugging.

Bœnninghausen gives us the following list of anti-sycotic remedies, which he divides, according to their greater or less similarity to sycosis, into four classes, namely:

First. Apis, Ars., Graph., Mezer., Phosph., Selen.

Second. Calc.-c., Caustic., Ferr., Jod., Puls., Sil.

Third. Anac., Ant.-crud., Bar., Bell., Carb.-an., Carbo-veg., Chin., Euphr., Hep., Kali-c., Phosph.-ac., Plat., Plumb., Sabad., Spig.

Fourth. Lach., Lyc., Nitr.-ac., Rhus-tox., Sep., Staph.

Since then some other remedies have been added, as Antimonium-tartaricum, Clematis, and especially Natrum-sulphuricum. The latter is highly praised as an anti-sycotic by Dr. Kent, and in his lecture on this remedy he also makes the very interesting statement that chronic bronchial affections almost always need anti-sycotics for their cure.

At last permit me to say a few words about vaccination, the third way sycosis is supposed to enter the human organism. If small-pox causes sycosis, vaccination may also do so. The occurrence of acute disturbances, assuming sometimes even a dangerous form, following the insertion of the vaccine virus into the system, seems to give us an affirmative answer. Whether, however, sycosis will follow after every vaccination is a question. Vaccine is but a modification—a potency, if you please—of variola virus, and the time of its protection against variola is certainly limited. Now, do individuals who have suffered severely of the vaccine virus ever acquire small-pox if exposed to it, even if not revaccinated? If experience would give us a negative answer, then, perhaps, we can conclude that vaccination made them sycotic.

The question, whether we, by vaccination, make persons sycotic, in other words, cause them to become cripples for their lifetime, is certainly a vital one.

Another interesting subject for settlement would also be the following: Can a person with latent sycosis, even if not vaccinated, have small-pox? And again, are not the persons with whom vaccination will not work sycotic, and therefore, *per se*, protected from small-pox?

Now, gentlemen, I hope I have given you something for discussion, which has been the sole purpose of this paper.

Dr. Hermance asked whether a case where crippled hands followed vaccination would be due to sycosis.

Dr. Biegler said: "The consequences of vaccination seem to depend upon the source of the vaccine virus. If this were taken from a sycotic person, sycosis might be transferred just as well as syphilis. Bœnninghausen wrote at a time when vaccination was done from arm to arm. He remembered only too well the time during the late Civil War, when syphilis was spread gratuitously by means of vaccination from arm to arm, and a good many vaccinations were none at all, as a good many times only small pustules appeared that dried up on the ninth day."

Dr. Hussey thinks that some children are not affected by vaccine virus at all; this may be taken as an evidence not that they are sycotic, but, on the contrary, perfectly healthy. He mentions, as an example, some children of his acquaintance who did not respond to the vaccination, nor were they ever affected by scarlet fever or measles, even after having been exposed to these diseases.

Dr. Clapp mentions that old constitutional symptoms, not belonging to sycosis at all, may be brought out by vaccination.

Dr. Stowe has found that lymph which had been recommended as the purest contained pus and blood corpuscles.

Dr. Biegler has been told by a physician, who has been studying closely the subject of vaccination, that he never found a case of diphtheria in non-vaccinated children, and this has influenced him so that he would not vaccinate a child until six to seven years old, if at all.

Dr. Stowe, when in Fall River, saw several cases of sycosis; all were English women and had fig-warts about their genitals. In one of them the base of the condylomata suppurated and was surrounded by a furrow, which undermined it, as it were. Silicea, two doses, cured in three to four months the local and constitutional symptoms. He is opposed to any kind of vaccination, even that performed with bovine virus, for this reason: The small-pox virus with which the heifer is inoculated is taken from a human being and will carry with it all the chronic poisons that this body may contain through the blood and body of the animal. The latter may not be affected at all by these poisons, but they will not be extinguished by their course through the animal, and their dynamis must be carried with the bovine virus to the human system into which this virus is introduced during the performance of vaccination.

If nothing else will come out of this discussion, it will certainly bring before us the serious question, whether we shall or shall not vaccinate. Gentlemen, we must sooner or later arrive at the point when compulsory vaccination will be abandoned, and instead of it, pure hygienic measures be adopted for the prevention of small-pox.

Dr. Hussey—We as homœopaths must make a difference between potentization and dilution as going on when the small-pox virus traverses the body of the cow. It is the same as in Pasteur's experiments with bacteria, when inoculating one glass from the other; he would not call that potentization, where it even is no attenuation at all.

Dr. Voak was the first physician in his place who refused to vaccinate with humanized vaccine virus; never thought of the danger of vaccinating with bovine virus until his attention was called to it to-day.

Dr. Clapp had a similar experience to Dr. Voak. When he commenced to vaccinate with bovine virus, the people were opposed to it on account of greater cost, but submitted gladly after several deaths from the effect of vaccination with humanized virus had occurred.

Dr. Biegler gave, as an example how thoroughly a contagions disease might be prevented from spreading by proper sanitary measures, the following experience: While he was a member of the Board of Health of Rochester, about ten years ago, the health officers reported to him at six P. M. a case of small-pox in a shop where second-hand clothing and similar articles were sold. He told the health officer to take the patient, who was the owner of the shop, to the pest-house at once and then take everything in the house that was combustible and burn it, and then to ventilate thoroughly the whole house. All this was promptly done the same evening and so there was no small-pox in Rochester. The patient sued the city for damages, and he does not know what became of it.

Dr. Stowe related the following occurrence that happened in the place where he resided at the time. There was a smallpox case in town; a young, ambitious physician inoculated a heifer from this patient, and then vaccinated with the virus of this animal about twenty-eight persons, all of whom got the small-pox.

A vote of thanks was tendered to Dr. Carr for his sumptuous repast; and as subject for discussion at the next meeting, Sycosis was again selected.

The meeting then adjourned to meet at Dr. Hawley's office in Syracuse on the third Thursday of March, 1887.

